# Subscapular elastofibrolipoma treated with marginal resection: two case reports 

**DOI:** 10.1186/s13256-022-03522-4

**Published:** 2022-08-02

**Authors:** Parviz Mardani, Hooman Kamran, Nazanin Ayare, Reza Shahriarirad, Pardis Shahabinejad, Bita Geramizadeh, Masoud Vafabin

**Affiliations:** 1 Thoracic and Vascular Surgery Research Center, Shiraz University of Medical Science, Shiraz, Iran; 2 Department of Surgery, Shiraz University of Medical Sciences, Shiraz, Iran; 3 Student Research Committee, Shiraz University of Medical Sciences, Shiraz, Iran; 4 School of Medicine, Shiraz University of Medical Sciences, Shiraz, Iran; 5 Shiraz Transplant Research Center (STRC), Shiraz University of Medical Sciences, Shiraz, Iran; 6 Department of Pathology, Shiraz University of Medical Sciences, Shiraz, Iran

**Keywords:** Elastofibroma dorsi, Elastofibrolipoma, Fibrolipoma, Neoplasm, Scapula

## Abstract

**Background:**

Elastofibroma dorsi is a rare benign tumor of soft tissue, typically under the lower angle of the scapula. Its specific location and distinctive clinical symptoms can provide enough information for diagnosis. Nevertheless, pathological confirmation by biopsy may be needed to rule out other malignancies.

**Case presentation:**

Here, we present two cases of 63-year-old and 49-year-old female Asian patients who came to us with the chief complaint of pain and bulging in their shoulders. Both patients had rubbery and mobile masses. Also, shoulder movements were not restricted in the examination; however, the patients expressed pain during movements. Computed tomography scans were compatible with the diagnosis of elastofibroma dorsi. Surgical excision was performed for both cases owing to the symptomatic nature of the masses, and histopathological findings confirmed the diagnosis.

**Conclusion:**

Elastofibroma dorsi is a benign pseudotumor presenting with an uncomfortable feeling in the shoulder with movement in older females. In typical symptom-free cases of elastofibroma dorsi, observation is sufficient, while in symptomatic patients or if there is suspicion of malignancy, complete resection with marginal resection is the treatment of choice.

## Background

Elastofibroma dorsi (EFD) is a rare, slow-growing, nonencapsulated, benign tumor with soft tissue origin [1]. The prevalence of EFD in computed tomography (CT) reports is 2–2.73 and 24% in autopsies [2, 3]. These rates of incidence show that most patients are asymptomatic [4, 5]. The disease was first described in 1961 by Jarvi and Saxen [6]. The pathogenesis of elastofibroma is still unknown; however, repeated microtraumas between the chest wall and the scapula can be a source of excess elastin production and collagen degeneration, which could explain the origin of the disease [7, 8]. This etiology has been supported by higher incidence, especially among those working in hard manual labor, although it can also happen in individuals who have never worked in hard manual labor jobs [9–11]. It occurs mostly in the muscles attached to the inferior angle of the scapula, beneath the rhomboid major and latissimus dorsi muscles of older women in their fourth to sixth decades [9, 10]. Whether it is a true neoplasm or not is still in question, since EFD behaves like a malignant lesion attaching to muscle, periosteum of ribs, and scapula [12, 13]. EFD is usually unilateral but can happen bilaterally in 10% of cases [11]. CT imaging and magnetic resonance imaging (MRI) have a role in identifying lesions but can be mistaken for neoplasms. Thus, excisional biopsy is preferred for diagnosis [4, 10]

This study describes two patients: one with a case of bilateral elastofibrolipoma and the other with unilateral fibrolipoma, presenting with shoulder bulging and pain, diagnosed with typical histological appearance. Surgical excision was performed owing to the symptomatic nature of the masses in our cases.

## Case presentation

### Case 1

**Fig. 1:**
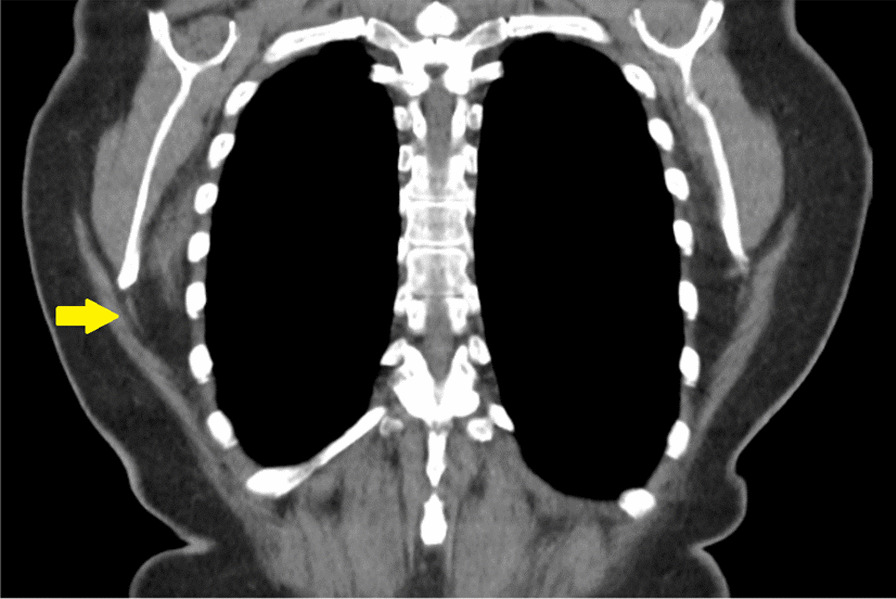
Computed tomography of a case with elastofibrolipoma (yellow indicator)

A 63-year-old female Asian patient was referred to our hospital with a chief complaint of bilateral shoulder bulging and, also, right-sided shoulder pain, especially during shoulder movements. On physical examination, bilateral well-defined masses were detected in the subscapular area, of which the right-sided mass was larger. Both masses were rubbery and mobile. Also, shoulder movements were not restricted in the examination; however, the patient expressed pain during movements. Contrast material-enhanced CT demonstrated a soft-tissue mass. The borders of the mass were indistinct, but adjacent fat planes were preserved. The majority of the mass had an attenuation similar to that of the adjacent skeletal muscle; however, scattered microscopic linear fat attenuation was seen centrally throughout the mass. There were no nodular areas of contrast enhancement, no calcification, and no adjacent osseous destruction. No tissue sampling or further imaging was deemed necessary for diagnosis, since these were classic imaging findings and the location was typical for elastofibrolipoma. Figure 1 shows the CT scan of our patient.

Surgical excision was performed owing to the symptomatic nature of the mass at the right side. In lateral position, under general anesthesia, the chest wall skin, fascia, and muscles were opened, and an 8 × 10 cm mass was completely excised. The resection was done until the margin where the rubbery consistency of the mass was palpated. It is possible that some parts of the surrounding fibrous tissue remained. A Jackson–Pratt (JP) drain was inserted, and subsequently, the chest wall was closed. Also, the mass was sent for pathological evaluation.

**Fig. 2:**
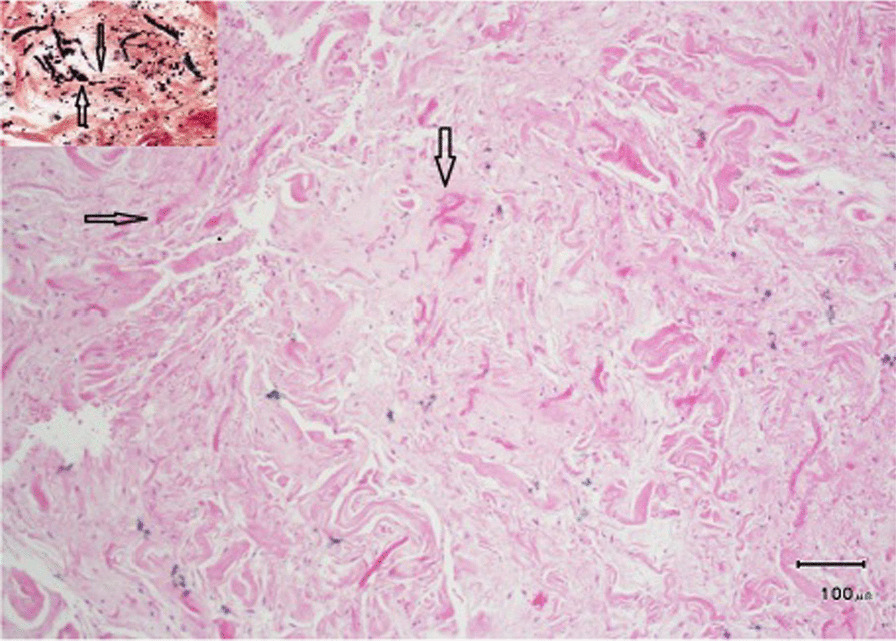
Pathological section of the mass excised from the chest wall of a 63-year-old male, in favor of elastofibroma

The mass was halved during resection, and two irregular, semi-ovoid, gray-yellowish, rubbery, solid masses were received by the pathologist: one was 5.5 × 4 × 2.5 cm with attached soft tissue of 5.5 × 2 × 1 cm, and the other one was 3 × 3 × 2 cm with attached soft tissue of 3 × 3 × 1 cm. The cut section of the larger mass showed a white-yellowish solid area, and the cut section of the smaller mass showed a homogeneous yellowish area without hemorrhage or necrosis. Microscopic examination showed paucicellular tissue with thick densely eosinophilic elastin bands, which were positive with Verhoeff elastin stain (Fig. 2). This histologic picture is typical for elastofibroma, and the diagnosis was elastofibrolipoma. The patient has been symptom-free for 14 months since the operation.

### Case 2

**Fig. 3:**
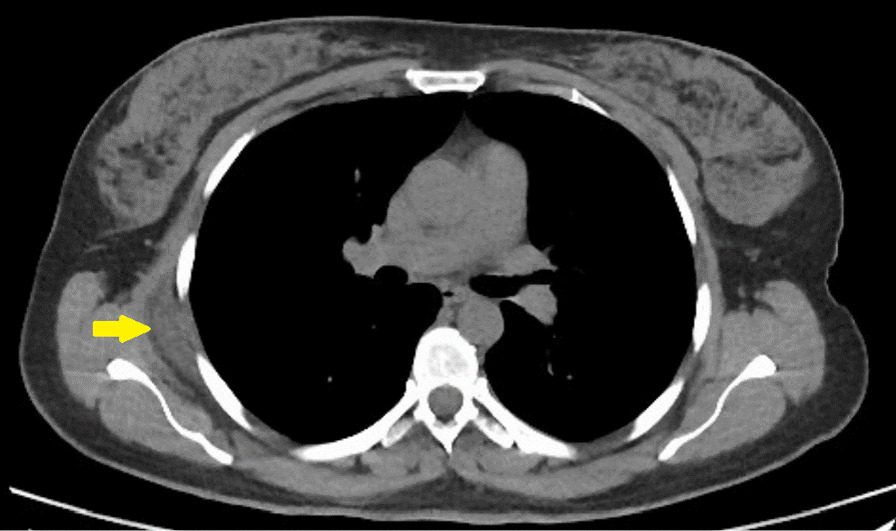
Computed tomography of a case with unilateral fibrolipoma (yellow indicator)

A 49-year-old female Asian patient with a past medical history of hypothyroidism who suffered from unilateral shoulder bulging and on–off right shoulder pain was referred to our hospital. The pain was worsened during shoulder movements. On physical examination, right-sided subscapular mass was palpated, which was rubbery and mobile. In addition, although the patient had pain during the examination of the shoulder movements, but no movement restriction. The CT findings was similar to those of the previous case. Figure 3 shows the CT scan of our patient.

**Fig. 4:**
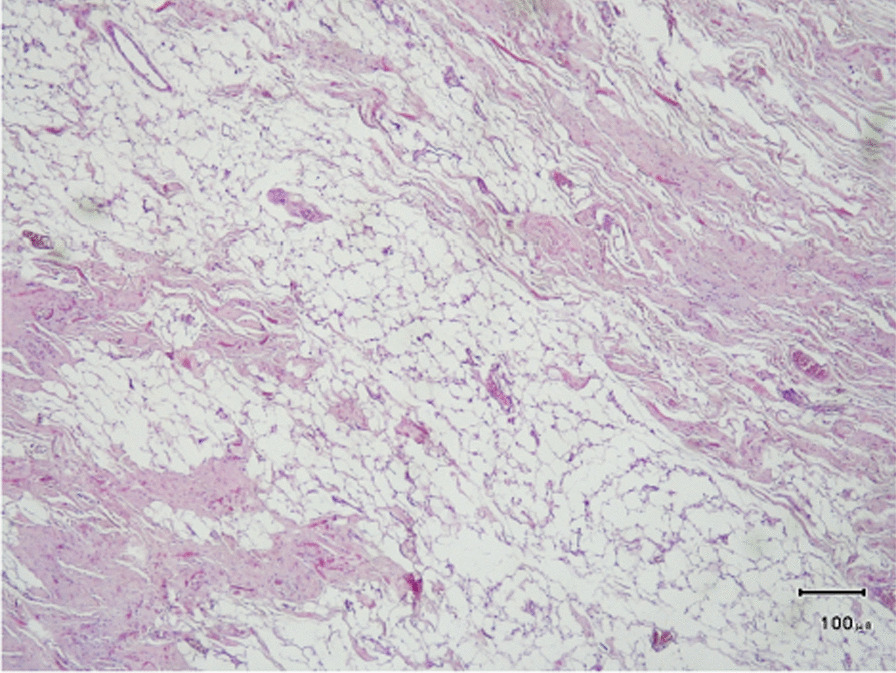
Pathological section of the mass excised from the chest wall of a 49-year-old female, in favor of elastofibroma

The surgical excision was the same as the previous case. Macroscopic examination revealed a gray, rubbery mass with irregular borders of 9 × 5 × 3.5 cm size. Also, cut sections showed white-yellowish solid areas. Microscopic examination showed a low-cellular tumor with some thick elastin fibers, which were positive with Verhoeff elastin stain. No atypia was present (Fig. 4). The diagnosis was reported fibrolipoma. The patient has been symptom-free 15 months since the operation.

## Discussion

Elastofibroma dorsi is a rare benign tumor originating from soft tissue described for the first time in 1961 by Jarvi and Saxen [1, 6]. It is generally found beneath the rhomboid major and latissimus dorsi muscles in the lower pole of the scapula, but it can also be located at unusual sites such as ischial tuberosity, olecranon, thoracic wall, and axilla [9, 10, 14]. It should be considered as a differential diagnosis in patients who feel pain and discomfort in the scapula. Other benign and malignant lesions of soft tissue of the chest wall, such as sarcoma, lipoma, fibroma, aggressive fibromatosis, desmoid tumor, and hemangioma, should be considered in these patients [10]. As the cases in our report, patients are usually elderly, and female patients make up the vast majority of this population with a sex ratio that fluctuates between 5:4 and 13:1. The reason for the higher prevalence among women is still unclear [9, 14, 15].

Several pathogenic theories have been proposed. The cause is still unknown, but it is suggested that repetitive frictions between the lower part of the scapula and thoracic wall cause microtraumas, leading to reactive hyperproliferation of elastic fibers within a stroma of collagenous adipose tissue [8]. As a result, elastofibroma dorsi typically occurs in the infrascapular and subscapular areas, but it is not exclusive to these parts. In addition to this mechanical explanation, there are studies in the literature that support genetic predisposition theory. Around 38% of the 170 cases in the largest series in the literature occurred within the same family lines [4]. There is also another genetic hypothesis that proposes that EFD is caused by varying degrees of gene hypo- or hyperexpression on different chromosomes. Some researchers have also described how EFD develops as a result of vascular insufficiency and relative ischemia in the scapular region. This theory has received little attention and was mentioned only as a reference hypothesis thus far. Other factors that may contribute to its pathogenesis include elastotic collagen degeneration, and abnormal elastotic fibrinogenesis [16–19].

Patients mainly present with bilateral lesions, which are asymptomatic in 50% of cases. Nevertheless, in case of being symptomatic, which depends on its size and location, it usually presents with pain exacerbation and discomfort, especially with shoulder movement and slow-growing swelling in infrascapular region, such as the cases presented in this article [9, 11].

Elastofibroma dorsi has typical imaging and histological findings. CT scans or magnetic resonance imaging (MRI) reports can show more specific findings. In particular, MRI is capable of showing expansile, solid, non-encapsulated, and heterogeneous tumor. Entrapped fat within a predominantly fibrous mass is demonstrated on T1- and T2-weighted sequences by predominant signal in comparison with muscles, which is an indicator of fibrous tissue and is typically intermingled with hypersignal lines representing fat tissue [8, 20].

When the lesion appears as heterogeneous, indistinct, and non-encapsulated infra- or subscapular soft-tissue mass, isoattenuating to muscle (fibrous tissue) with strands of fat attenuation, CT can be diagnostic. However, the lesion can also appear homogeneous when small [21–23]. These findings can provide enough information for diagnosis.

In case of suspicion of malignancy or the presence of symptoms, confirmation by biopsy is needed. Excisional biopsy is the preferred method of confirming the diagnosis and excluding malignancy [9–11]. The hyperproliferation of fibroblastic tissue distinguishes EFD. Tumor histopathology reveals collagen and coarse enlarged elastic fibers [13]. CT scan was performed in both cases, showing heterogeneous masses supporting the diagnosis. Postoperative histopathologic findings were also compatible with elastofibroma dorsi.

Since there are no reported malignant transformations, surgery in asymptomatic patients is not indicated, and observation of the patient is enough. In symptomatic patients, surgical excision with marginal resection is sufficient. Radiotherapy can be used if lesions are unresectable owing to their location, if the lesions recur because of incomplete resection, or if patients are at high medical-anesthetic risk [10].

## Conclusion

Elastofibroma dorsi is a benign pseudotumor presenting with an uncomfortable feeling in the shoulder with movement in older females. In typical symptom-free cases of elastofibroma dorsi, complementary studies are necessary, and observation is sufficient. In symptomatic patients or if there is suspicion of malignancy, complete resection with marginal resection is the treatment of choice.

## Data Availability

All data regarding this case report has been reported in the manuscript. Please contact the corresponding author in case of requiring any further information.

## Abbreviations

CT: Computed tomography
JP: Jackson–Pratt
MRI: Magnetic resonance imaging
